# Liposarcoma masquerading as an inflammatory pseudotumor: a case report

**DOI:** 10.1186/s13256-016-0858-y

**Published:** 2016-03-18

**Authors:** Jessica J. H. Reagh, Robert P. Eckstein, Christina I. Selinger, Justin Evans, Sandra A. O’Toole, Anthony J. Gill

**Affiliations:** 1 Department of Anatomical Pathology, Royal North Shore Hospital, Pacific Highway, St Leonards, NSW 2065 Australia; 2 Tissue Pathology and Diagnostic Oncology, Royal Prince Alfred Hospital, Building 94 Missenden Road, Camperdown, NSW 2050 Australia; 3 Department of Surgery, Royal North Shore Hospital, Pacific Highway, St Leonards, NSW 2065 Australia; 4 University of Sydney, Sydney, NSW 2006 Australia

**Keywords:** Benign lipomatous tumor, Liposarcoma, MDM2

## Abstract

**Background:**

Distinguishing an atypical lipomatous tumor/well-differentiated liposarcoma from a benign lipomatous tumor on morphology alone can be difficult and there is an established role for MDM2 fluorescent *in situ* hybridization studies in making this differential diagnosis. There is no literature on the role for MDM2 fluorescent *in situ* hybridization studies in distinguishing between a well-differentiated liposarcoma with extreme fibrosis and a fibrosing inflammatory pseudotumor.

**Case presentation:**

We report the case of a 76-year-old Australian woman initially diagnosed by an excision biopsy with a retroperitoneal fibrosing inflammatory pseudotumor. She was then diagnosed 5 years later with a pleomorphic undifferentiated sarcoma. Upon review of the original resection specimen, we were able to show that the tumor demonstrated MDM2 amplification. MDM2 amplification was also present in some adjacent bland adipose tissue, and also in the tumor recurrence as a pleomorphic undifferentiated sarcoma.

**Conclusion:**

Taken together, our findings provide strong evidence that the original tumor was a misdiagnosed well-differentiated liposarcoma with extreme fibrosis, and the pleomorphic undifferentiated sarcoma represented a recurrence of the same tumor with dedifferentiation.

## Background

The distinction between atypical lipomatous tumor/well-differentiated liposarcoma (ALT/WDLPS) and benign lipomatous tumors can be difficult, and fluorescent *in situ* hybridization (FISH) studies for MDM2 amplification have an established role in the pathological differential diagnosis [1–8]. Distinguishing between WDLPS with extreme sclerosis and fibrosing inflammatory pseudotumor can be equally problematic, but FISH studies for MDM2 are infrequently used in this setting. We present a case of low grade liposarcoma initially misdiagnosed as a fibrosing retroperitoneal pseudotumor and discuss the utility of MDM2 FISH in establishing a definitive tissue diagnosis.

## Case presentation

**Fig. 1:**
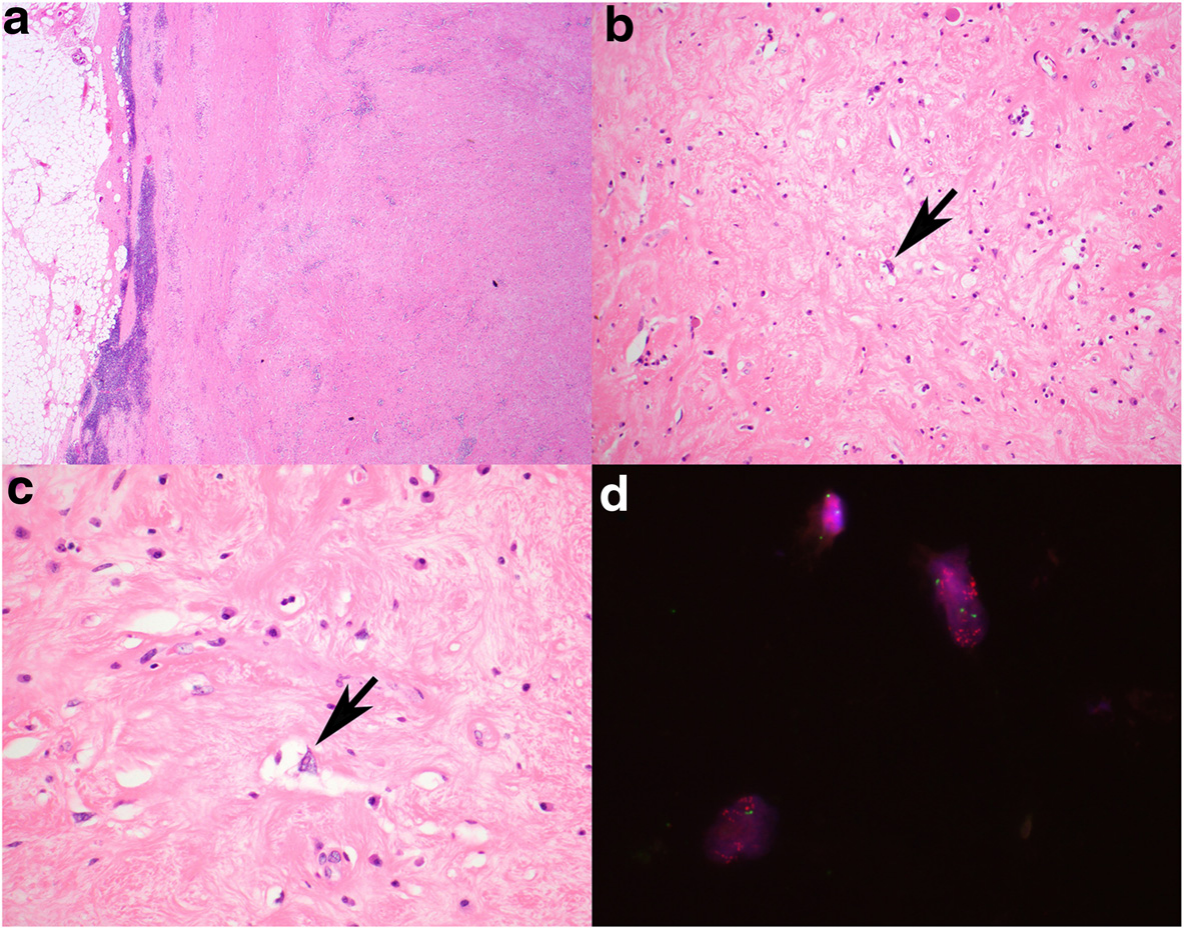
Representative photomicrographs from the initial resection specimen. **a** The tumor was relatively well circumscribed, with scattered lymphoid aggregates noted at the edge. **b** There was extensive fibrosis with scattered inflammatory cells and occasional enlarged cells with vague vacuolation (*arrow*). **c** Upon review, some of these atypical cells (*arrow*) showed cytoplasmic vacuolation raising the possibility of a lipoblast. **d** Fluorescent *in situ* hybridization studies demonstrated amplification of MDM2 (*red signal*) compared to the chromosome 12 centromere probe (*green signal*). Original magnifications **a** 20×, **b** 100×, **c** 400×, **d** 1000×

Our patient was a 76-year-old Caucasian Australian woman who originally presented over 5 years ago with urinary symptoms initially suggestive of recurrent urinary tract infection. At that time, she had an unremarkable past medical history of mild hypertension with no previous hospitalizations and did not smoke cigarettes. She underwent a work-up for the ongoing dysuria and urgency symptoms and imaging demonstrated a high-density retroperitoneal tumor with some fat density around her right inguinal canal. This tumor was surgically resected. On gross examination, the retroperitoneal tumor was 10 cm in diameter with a lobulated contour and a pale whorled cut surface reminiscent of uterine leiomyoma. On histological examination, there was widespread, dense fibrosis, with a variable chronic inflammatory infiltrate rich in plasma cells that was most prominent at the periphery of the tumor (Fig. 1a). The tumor was hypocellular and densely fibrotic, composed predominantly of bland stromal cells with morphology suggestive of fibroblasts (Fig. 1b, c). Very occasional mildly atypical larger cells were noted and some were vacuolated (Fig. 1b, c), but no diagnostic lipoblasts were identified. The fat from her right inguinal canal was composed of mature adipose tissue with extensive fat necrosis. Again, no lipoblasts were identified. Immunohistochemistry demonstrated that the tumor was negative for S100, CD34, cKit, desmin, and anaplastic lymphoma kinase (ALK), and there was no increase in immunoglobulin (Ig)G4-positive plasma cells. Although there was some uncertainty, the retroperitoneal tumor was diagnosed as an inflammatory pseudotumor in fibrosing stage (IgG4-negative variant). The area of fat necrosis in her right inguinal canal was considered benign.

**Fig. 2:**
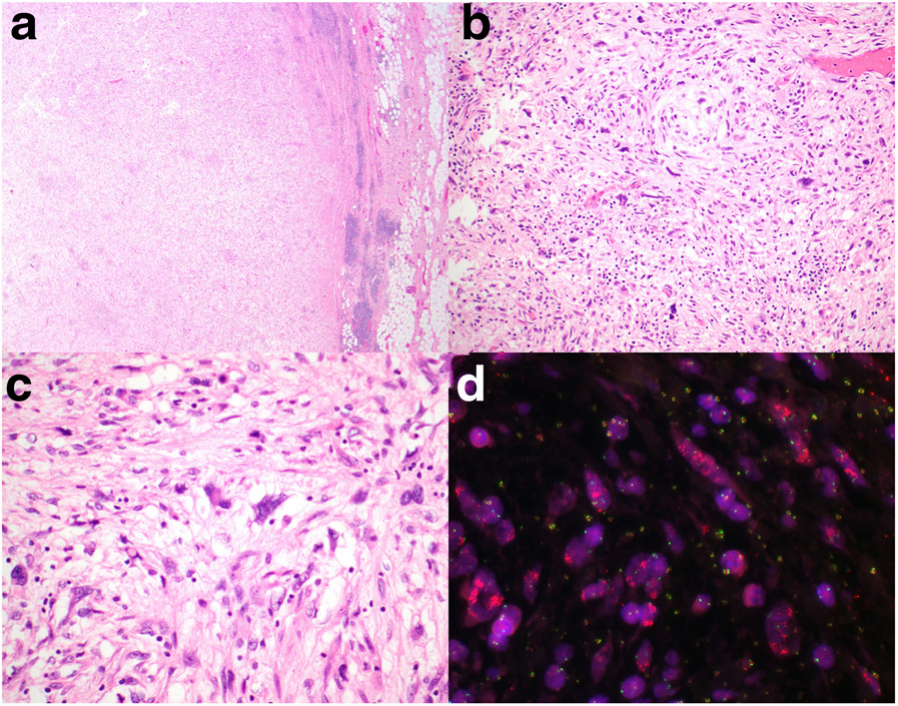
Representative photomicrographs of the tumor recurrence. **a** The tumor was well circumscribed and there was a peripheral rim of lymphoid aggregates. **b** At intermediate power, the tumor demonstrated marked nuclear atypia with morphology reminiscent of a pleomorphic undifferentiated sarcoma. **c** Very occasional cells demonstrated cytoplasmic vacuolation, morphologically suggestive of an origin from the dedifferentiation of liposarcoma. **d** Fluorescent *in situ* hybridization studies demonstrated amplification of MDM2 (*red signal*). Original magnifications **a** 20×, **b** 100×, **c** 400×, **d** 600×

Our patient re-presented 5 years later with similar urinary symptoms and imaging demonstrated a retroperitoneal tumor at the site of the previous excision. She underwent *en block* excision with a right hemicolectomy. The excised tumor was centered in the deep mesocolon. Again the tumor was lobulated and 10 cm in diameter with a somewhat pale fleshy cut surface. An incomplete rim of chronic inflammation was again noted (Fig. 2a) but the recurrent tumor was composed of poorly differentiated pleomorphic cells including bizarre forms (Fig. 2b, c). That is, the tumor demonstrated the morphology of a pleomorphic undifferentiated sarcoma. Despite the marked pleomorphism, mitoses were infrequent (less than 1 per 10 high-power fields). Again, occasional vacuolated cells were present (Fig. 2c) but classic lipoblasts were not identified.

Immunohistochemistry again demonstrated that the neoplastic cells were negative for S100, CD34, cKit, desmin, and ALK. Given the identical location and presence of a rim of inflammatory cells it was clear that the recurrence represented dedifferentiation of the primary tumor. Upon review of both cases, the possibility of dedifferentiated liposarcoma was considered. The primary tumor, the bland adipose tissue resected from the inguinal canal at the time of surgery, and the recurrent tumor all underwent FISH testing, which demonstrated MDM2 amplification in all three specimens (Figs 1d and 2d). Therefore a final diagnosis of ALT/WDLPS with subsequent dedifferentiation and recurrence as a dedifferentiated liposarcoma was made.

## Discussion

The demonstration of amplification of MDM2 with FISH has proven to be a robust and reliable method of differentiating ALT/WDLPS from benign lipomatous tumors [1–8]. For example, Kimura *et al*. found MDM2 amplification in 98 % (48 of 49) of ALT/WDLPS but in no benign adipose tumors [1]. Similarly Weaver *et al*. demonstrated MDM2 amplification in 100 % of ALT/WDLPS (13 out of 13) and dedifferentiated liposarcomas (14 out of 14) [2]. Of note MDM2 amplification has been consistently demonstrated in areas of WDLPS with minimal cellular atypia, indicating that the presence of MDM2 amplification even in cytologically bland adipose tissue can be considered *prima facie* evidence of ALT/WDLPS [2]. That is, if MDM2 FISH studies had been available and were performed prospectively in our case at first presentation in 2010, a diagnosis of ALT/WDLPS could have been justified despite the bland cytology.

There is also emerging evidence that the presence of MDM2 amplification in an otherwise undifferentiated sarcoma is strong evidence that the tumor has arisen from dedifferentiation of a liposarcoma. For example, in Le Guellec *et al*. [3] reported that tumors that would otherwise be classified as pleomorphic undifferentiated sarcoma, but that demonstrated MDM2 amplification, had similar clinical characteristics, morphology, genomic profile, and outcome to conventional dedifferentiated liposarcoma and were significantly less aggressive than pleomorphic undifferentiated sarcomas without MDM2 amplification. Our case, which provides clear evidence of a pleomorphic undifferentiated sarcoma arising from a lower grade tumor with MDM2 amplification, further supports this finding.

## Conclusion

To exclude ALT/WDLPS, it has been recommended that MDM2 FISH studies be performed in any atypical adipose tumors, including any adipose tumor with equivocal cytological atypia or cytologically bland tumors with recurrence, large size, deep location, and/or retroperitoneal location [7]. This case demonstrates that there may also be a role for MDM2 studies to exclude a diagnosis of WDLPS with extreme sclerosis in patients apparently presenting with a retroperitoneal inflammatory pseudotumor.

## Consent

Written informed consent was obtained from the patient for publication of this case report and accompanying images. A copy of the written consent is available for review by the Editor-in-Chief of this journal.
